# The eNAMPT/TLR4 inflammatory cascade drives the severity of intra-amniotic inflammation in pregnancy and predicts infant outcomes

**DOI:** 10.3389/fphys.2023.1129413

**Published:** 2023-06-20

**Authors:** Mohamed Ahmed, Nancy G. Casanova, Nahla Zaghloul, Akash Gupta, Marisela Rodriguez, Ian R. Robbins, Carrie L. Kempf, Xiaoguang Sun, Jin H. Song, Vivian Reyes Hernon, Saad Sammani, Sara M. Camp, Alvaro Moreira, Chaur-Dong Hsu, Joe G. N. Garcia

**Affiliations:** ^1^ Departments of Pediatrics, University of Arizona Health Sciences, Tucson, AZ, United States; ^2^ Department of Medicine, University of Arizona Health Sciences, Tucson, AZ, United States; ^3^ Department of Pediatrics, UT Health San Antonio, Long School of Medicine, San Antonio, TX, United States; ^4^ Department of Obstetrics and Gynecology, University of Arizona Health Sciences, Tucson, AZ, United States

**Keywords:** intra-amniotic inflammation (IAI), eNAMPT, damage-associated molecular pattern protein (DAMP), preterm birth, bronchopulmonary dysplasia (BPD), mAb

## Abstract

**Introduction:** Intra-amniotic inflammation (IAI) or chorioamnionitis is a common complication of pregnancy producing significant maternal morbidity/mortality, premature birth and neonatal risk of chronic lung diseases such as bronchopulmonary dysplasia (BPD). We examined eNAMPT (extracellular nicotinamide phosphoribosyltransferase), a critical inflammatory DAMP and TLR4 ligand, as a potential therapeutic target to reduce IAI severity and improve adverse fetal/neonatal outcomes.

**Methods:** Blood/tissue samples were examined in: 1) women with histologically-proven chorioamnionitis, 2) very low birth weight (VLBW) neonates, and 3) a preclinical murine pregnancy model of IAI. Groups of pregnant IAI-exposed mice and pups were treated with an eNAMPT-neutralizing mAb.

**Results:** Human placentas from women with histologically-proven chorioamnionitis exhibited dramatic NAMPT expression compared to placentas without chorioamnionitis. Increased *NAMPT* expression in whole blood from VLBW neonates (day 5) significantly predicted BPD development. Compared to untreated LPS-challenged murine dams (gestational day 15), pups born to eNAMPT mAb-treated dams (gestational days 15/16) exhibited a > 3-fold improved survival, reduced neonate lung eNAMPT/cytokine levels, and reduced development and severity of BPD and pulmonary hypertension (PH) following postnatal exposure to 100% hyperoxia days 1–14. Genome-wide gene expression studies of maternal uterine and neonatal cardiac tissues corroborated eNAMPT mAb-induced reductions in inflammatory pathway genes.

**Discussion:** The eNAMPT/TLR4 inflammatory pathway is a highly druggable contributor to IAI pathobiology during pregnancy with the eNAMPT-neutralizing mAb a novel therapeutic strategy to decrease premature delivery and improve short- and long-term neonatal outcomes. eNAMPT blood expression is a potential biomarker for early prediction of chronic lung disease among premature neonates.

## 1 Introduction

Intra-amniotic inflammation/infection (IAI) and chorioamnionitis are implicated in ∼30% of preterm births (defined as <37 weeks) and the majority of extreme preterm births (<28 weeks) (Blencowe et al., 2012; Kinney et al., 2012; Musilova et al., 2021). IAI is defined as an inflammatory or infectious disorder involving a combination of amniotic fluid, placenta, fetus, fetal membranes, or decidua (Obstet Gynecol, 2017). The bacterial migration from the lower genital tract across the cervical barrier to penetrate chorioamniotic membranes and enter the amniotic fluid produces IAI and tissue inflammation (chorion, amnion, placenta, umbilical cord) resulting in the fetal inflammatory response syndrome (FIRS) (Gotsch et al., 2007; Yee et al., 2011). FIRS is defined by increased fetal cytokines (Gotsch et al., 2007), umbilical cord infection/inflammation, and vasculitis (Watterberg et al., 1996; Nguyen et al., 2015; Rogers and Hintz, 2016; Schieve et al., 2016; Cappelletti et al., 2020). IAI-induced FIRS and preterm births contribute to >70% of perinatal mortality in developed countries (Kinney et al., 2012) and to ∼1 million neonatal deaths world-wide annually (Benjamini and Hochberg, 1995; Kinney et al., 2012). Importantly, surviving preterm infants remain at risk for long-term morbidity and life-long disabilities (Behrman and Butler, 2007; Rogers and Hintz, 2016) including chronic lung diseases such as bronchopulmonary dysplasia (BPD) and pulmonary hypertension (PH). Antimicrobial therapies have improved extreme preterm infant survival (∼50%) (Dempsey et al., 2005) but have been ineffective in preventing IAI-associated morbidities including preterm delivery, risk of respiratory distress syndrome, sepsis, intra-ventricular hemorrhage, and necrotizing enterocolitis (Kenyon et al., 2013; Kahramanoglu et al., 2016; Kalikkot Thekkeveedu et al., 2017; Chao et al., 2018). Novel therapeutic approaches are needed to reduce the devastating morbidity associated with IAI in pregnancy.

Experimentally, IAI is induced by systemic injection of diverse pathogen-associated molecular pattern molecules (PAMPs) such as LPS, damage-associated molecular patterns (DAMPs) such as HMGB1, or live microorganisms. Via ligation of pathogen recognition receptors (PRRs), such as Toll-like receptors (TLRs), PAMPs, DAMPs and microbes produce the characteristic features of acute chorioamnionitis with neutrophil infiltration of chorion and amnion membranes and amniotic fluids (Bose et al., 2009; Hartling et al., 2012; Jobe, 2012). These events reflect innate immunity inflammatory cascade activation producing maternal-fetal inflammation of the decidua and amniochorion, NFkB-mediated oxidant/inflammatory gene expression and release of cytokines that elicit cervical ripening, fetal membrane rupture, myometrial activation and preterm delivery. Current anti-inflammatory therapeutic strategies for IAI include NF-kB inhibitors (Bhat et al., 2012), N-acetyl-cysteine (NAC) (Mourani et al., 2015), sulfasalazine, and small molecule inhibitors or mAbs targeting TNF-α and IL-1β inflammatory pathways (Hansen et al., 2010; Eriksson et al., 2015). Currently, there are no FDA-approved therapies for chorioamnionitis/IAI-induced inflammation, a major unmet need.

eNAMPT (extracellular nicotinamide phosphoribosyltransferase) is an upstream, proinflammatory cytozyme and novel DAMP that is a major ligand of the PRR, TLR4 (Camp et al., 2015). Blood eNAMPT levels serve as danger signals and are increased in response to bacterial/viral infection, hypoxia, and mechanical stress including cyclic stretch serving as an inflammatory biomarker in diverse human inflammatory disorders (Bime et al., 2019; Sun BL. et al., 2020; Sun X. et al., 2020; Bime et al., 2021). eNAMPT ligation of TLR4 elicits innate immunity responses, NF-kB transcriptional activities, and profound inflammatory lung injury and driving the severity of clinical and preclinical ARDS (Bermudez et al., 2022; Sammani et al., 2022), pulmonary hypertension (PH) (Sun X. et al., 2020; Ahmed et al., 2021), radiation-induced lung disease (Garcia et al., 2022a; Garcia et al., 2022b), lupus vasculitis (Tumurkhuu et al., 2023), and non-alcoholic hepatic fibrosis (Sun et al., 2023). Blood eNAMPT levels are elevated during pregnancy (Mazaki-Tovi et al., 2009; Pavlova et al., 2018; Musilova et al., 2021) but have limited specificity for the presence of chorioamnionitis. The current study examines the hypothesis that eNAMPT secretion into the maternal-fetal circulation significantly contributes to IAI and FIRS development and adverse maternal/infant outcomes. We assessed the eNAMPT/TLR4 inflammatory pathway as a druggable IAI target in pregnancy utilizing a humanized eNAMPT-neutralizing mAb in a murine model of IAI/chorioamnionitis-induced uterine and neonatal inflammation as well as premature birth. Our preclinical studies suggest that the attenuation of eNAMPT/TLR4 inflammatory signaling is a potentially novel therapeutic strategy to reduce maternal and fetal/neonatal morbidities and mortalities as well as prematurity in human pregnancies complicated by IAI/chorioamnionitis.

## 2 Materials and methods

### 2.1 Human pregnancy and neonatal subject cohorts

Demographic data of pregnant women groups and the human neonatal cohort are shown in Tables 1, 2.

**TABLE 1:** Clinical and Demographic Data in Two Pregnant Women Cohorts Assessed for Serum eNAMPT Levels.

Parameters	Subjects without chorioamnionitis (n = 27)	Subjects with chorioamnionitis (n = 40)	*p* value
Age group (mean+/-SD)	28.12 + 2.3	24.4 + 3.4	<0.05
Ethnicity (%white)	62%	58%	NS
BMI	27.12 + 32	28.34 + .42	NS
Primiparous %	26%	28%	NS
Smoking %	22%	18%	NS
Systolic BP (mmHg)	105.4 + 2.3	102.5 + 3.4	NS
Diastolic PB (mmHg)	70.5 + 2.1	72.4 + 2.5	NS
Fasting glucose (mg/dL)	74.12 + 2.3	78.5 + 2.8	NS
HR (beat/min)	82 + 5	96.4 + 5.2	<0.05
Creatinine (mg/dL)	62.7 + 1.8	66.5 + 2.1	NS
GAS (weeks)	38.9 + 0.1	34.5 + 1.2	<0.05
GAD (weeks)	38.9 + 0.1	35.5 + 1.4	<0.05
WBC count at admission	6.2 + 1.4	12.6 + 3.2	<0.05
PROM >18 h	11%	25%	<0.05
Delivery mode-Vaginal; C-section	74%; 26%	65%; 35%	NS
Live at birth	100%	100%	NS
Birth weight (kg)	3.2 ± 0.42	2.25 ± 0.33	<0.05
Apgar score <7 at 5 min	4%	5%	NS

BMI, body mass index; DBP, diastolic blood pressure; GAD, gestational age at delivery; GAS, gestational age at admission; Hb; Hct, hematocrit; HR, heart rate; SBP, systolic blood pressure.

**TABLE 2:** Clinical data in the very low birth weight (VLBW) human neonatal cohort.

Variable	Overall	No BPD	BPD	*p*-value^b^
	N = 36^a^	N = 18^a^	N = 18^a^	
Birthweight (grams)	1,165 (1,000, 1,342)	1,238 (1,158, 1,348)	1,045 (985, 1,248)	<0.02
Male sex	18 (50%)	8 (44%)	10 (56%)	0.50
Gestational age (weeks)	29.0 (28.0, 30.0)	29.0 (28.0, 30.0)	29.0 (28.0, 30.0)	>0.90
*NAMPT* expression in whole blood	3.46 (3.37, 3.54)	3.39 (3.36, 3.52)	3.47 (3.45, 3.54)	<0.037

^a^ Median (IQR) or Frequency %.

^b^ Wilcoxon rank sum test; Pearson’s Chi square; Wilcoxon rank exact sum test.

### 2.2 Reagents

All reagents were purchased from Sigma-Aldrich (St. Louis, MO), unless otherwise noted. The eNAMPT-neutralizing humanized mAb, ALT-100, was provided by Aqualung Therapeutics Corporation (Tucson, AZ) (Ahmed et al., 2021; Quijada et al., 2021; Sun et al., 2021). See Supplementary Methods for further details.

### 2.3 Immunohistochemistry (IHC) for NAMPT expression in human placentas

Chorio tissues were collected after birth from pregnant women with and without IAI. IHC staining for NAMPT was performed with anti-NAMPT (Bethyl Laboratories, Montgomery TX) as previously reported (Ahmed et al., 2021; Quijada et al., 2021; Garcia et al., 2022a). All studied cases of chorioamnionitis were pathologically classified as grade1 (Redline et al., 2003). See Supplementary Methods for additional details.

### 2.4 Murine model of intrauterine inflammation

Timed-pregnant C57BL6 mice (aged 8–10 weeks) were confirmed as day 0 of gestation (serial physical examinations, abdominal ultrasound). LPS (50 ug/mouse) was administered IP to pregnant mice at day 15 of gestation to produce placental, intrauterine and systemic inflammation (Fricke et al., 2018), a well-established IAI model (Kramer, 2011; Kamity et al., 2014) that results in severe IAI in 80%–90% of dams and fetal loss with premature abortion within 24–48 h (Wang et al., 2006; Kramer, 2011; Martinelli et al., 2012; Kamity et al., 2014). Treated dams group received two doses of eNAMPT mAb (10 ug/mouse IP) on day GD15 and GD16. See Supplemental Figure S1 and Supplementary Methods for additional details.

### 2.5 Ultrasound evaluation of fetal viability

The initial dam abdominal ultrasound was performed prior to LPS challenge to define the number of sacs in each uterine horn. The second ultrasound was performed 48 h after LPS administration to determine the total number of fetuses and viability in each dam. After birth, the number of surviving pups in each group was recorded to determine the survival rate.

### 2.6 Biomarker measurements

Measurements of serum, plasma and lung/uterine homogenate biomarker levels of IL-6, KC/IL-8, IL-1β, TNFα, and eNAMPT were measured utilizing meso-scale ELISA platform (Meso Scale Diagnostics, Rockville, MD) as we have previously described (Velten et al., 2010; Kamity et al., 2014; Bermudez et al., 2022).

### 2.7 Western blotting of tissue homogenate proteins

Protein expression of multiple proteins in uterine and lung homogenates was assessed by Western blotting as previously described (Ahmed et al., 2021; Garcia et al., 2022a; Garcia et al., 2022b).

### 2.8 Assessment of murine BPD and PH

Lungs from surviving pups exposed to hyperoxia (FiO2 85%, 14 days) were collected at 3 weeks of age for lung morphology studies and H&E staining as previously described (Quijada et al., 2021). Neonatal lung homogenates were examined for expression of eNAMPT, CD31, and SNAIL1 as indices of PH. Hemodynamic measurements of right ventricular systolic pressure (RVSP) and RV/S + LV ratios were evaluated 1–2 weeks post hyperoxia exposure as previously described (Ahmed et al., 2012; Chen et al., 2017; Sun X. et al., 2020). Murine echocardiography was performed as previously reported (Ahmed et al., 2021). See Supplementary Methods for additional details.

### 2.9 Quantification of micro-vessel density and vascular wall thickness

These studies were performed as described previously (Ahmed et al., 2012) with results expressed as the percentage of total vessel size. Percent wall thickness was calculated as (2 x wall thickness)/external diameter. See Supplementary Methods for additional details.

### 2.10 Microarray data analysis in neonates

*NAMPT* gene expression in day 5 blood from extreme preterm neonates (Pietrzyk et al., 2013) was examined as an early predictor of bronchopulmonary dysplasia (BPD) (Jobe and Bancalari, 2001). From a total of 20,697 genes, two *NAMPT* gene probes meeting the false discovery rate (FDR) threshold (<5%) were analyzed as an average (Kim et al., 2015). To demonstrate *NAMPT* expression as a predictor for development of BPD and not a surrogate of prematurity or a reflection of confounders such as lower gestational age, birthweight, or sex differences, a propensity score match was performed. Using a 1:1 nearest neighbor matching algorithm with distances based on logistic regression, we matched gestational age in weeks with a caliper of 0.2 of the standard deviation of the regression model using the MatchIt package in (Austin, 2011). See Supplementary Methods for additional details.

### 2.11 RNA sequencing of murine tissue samples

Following mouse uterine and heart RNA extraction, and RNA QC and library construction performed. Reads were mapped using Hisat2 v2.05 and assembled by StringTie (v1.3.3b) as previously described (Gentleman et al., 2004; Ritchie et al., 2006; Tao et al., 2007; Trapnell et al., 2010). Differential gene expression was performed using DESeq2 (1.20. s0) (Benjamini and Hochberg, 1995). The adjusted *p*-value FDR was utilized to control for multiple testing error (Langmead and Salzberg, 2012). Enrichment analysis for Gene Ontology (GO) classification focused on biological process and pathway classification with KEGG and Reactome sources (Kanehisa et al., 2004). See Supplementary Methods for additional details.

### 2.12 Statistical analysis.

Continuous data were compared using non-parametric methods and categorical data by chi square test. Where applicable, standard one-way ANOVA was used and groups were compared using the Newman-Keuls test. Differences between groups were considered statistically significant when *p* < 0.05. Two-way ANOVA was used to compare the means of data from two or more different experimental groups. If significant differences were present by ANOVA, a least significant differences test was performed *post hoc*. Statistical tests were performed using GraphPad Prism version 7.00 for Windows (GraphPad Software, La Jolla, CA).

## 3 Results

### 3.1 Increased NAMPT uterine expression in human chorioamnionitis

**FIGURE 1:**
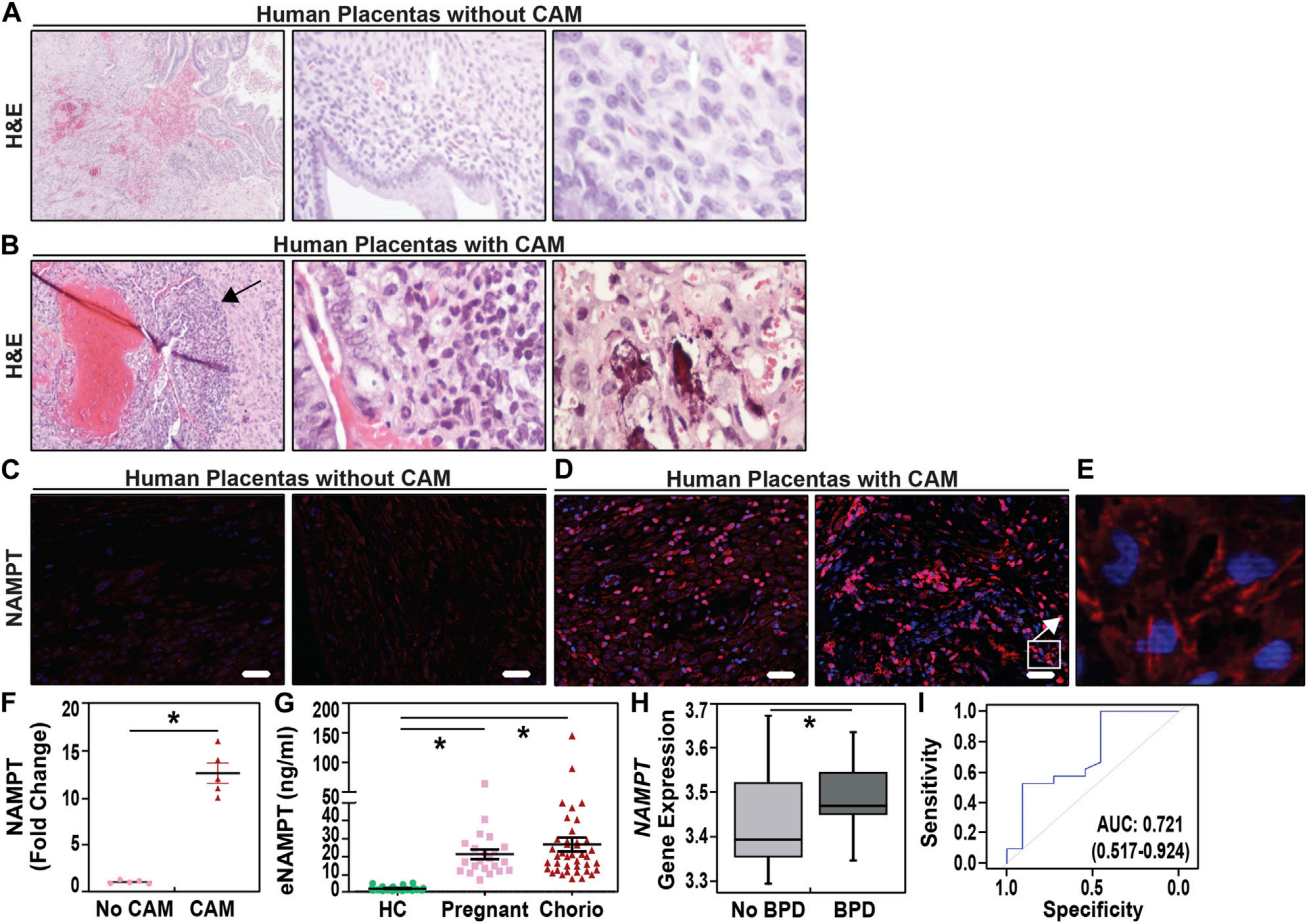
Increased uterine and plasma NAMPT expression in pregnant women with chorioamnionitis. **(A,B)**. Representative H&E staining images of placentas from normal term women (38–40 weeks) and women with acute chorioamnionitis. A band of neutrophils is observed in placentas from women with acute chorioamnionitis in the chorion (arrow). See Table 1 for clinical demographic details. H&E section magnifications are 10X, 20X, and 40X, left to right. **(C,D,E)**. Immunostaining of eNAMPT in human placental membranes with chorioamnionitis and without chorioamnionitis. eNAMPT expression, represented by red staining, was significantly increased in human placental membranes with acute chorioamnionitis as compared to those without chorioamnionitis (signals in red). A higher magnification image of a human placenta with acute chorioamnionitis is depicted in Panel E (100x). Scale bar = 100 μm. **(F)**. Scatter dot plot quantification of eNAMPT signal intensity as fold change when compared to placental membranes without chorioamnionitis (Scale bar = 100 μm, n = 5 placental membranes/group; four sections/placental membrane; mean ± SE, *p < 0.05). **(G)**. Plasma eNAMPT levels in pregnant women with chorioamnionitis (Chorio, N = 40) and without chorioamnionitis (pregnant, N = 22) compared to healthy non-pregnant female controls (HC, N = 6). Scatter dot plot shows mean ± SE. *p < 0.05. **(H)**. NAMPT gene expression in Day 5 whole blood from VLBW neonates who subsequently developed BPD (n = 62) and neonates without BPD (n = 35) *p < 0.0005. **(I)**. Assessment of the predictive capacity of NAMPT gene expression demonstrated a highly significant area under the receiver operating characteristic curve (AUC) of 0.721 with 95% confidence intervals.

Pregnant women were identified with chorioamnionitis based upon clinical presentation and placental histology. H&E staining (Figures 1A,B) and IHC evaluation of NAMPT expression (Figures 1C,D,E) in placental membranes from women with chorioamnionitis (n = 5) revealed significant inflammatory changes with neutrophil infiltration and focal necrosis (representative images in Figures 1A,B) and >10 fold increases in NAMPT staining compared to women without chorioamnionitis (n = 5) (Figure 1F).

Serum eNAMPT levels were studied in three female cohorts: healthy female controls (ages 25–35 yrs old, n = 6), pregnant women without IAI/chorioamnionitis (n = 27) and women with IAI/chorioamnionitis (n = 40) (Table 1). Cases with pathological verification of chorioamnionitis presented at GA at an earlier average gestational age of 34.5 + 1.2 weeks and delivered at an earlier gestational age of 35.4 + 1.4 weeks when compared with cases without pathological chorioamnionitis (35.1 + 1.4, 38.1 + .9 weeks, respectively). Figure 1G depicts the significant increases in serum eNAMPT levels observed in pregnant women (17.01 ± 2.2 ng/mL) and IAI women (19.96 ± 3.9 ng/mL) compared to non-pregnant female controls (1.49 ± 0.19 ng/mL). No significant difference in serum eNAMPT level between pregnant women with or without chorioamnionitis (*p* > 0.05).

### 3.2 Human *NAMPT* expression in blood predicts development of bronchopulmonary dysplasia (BPD) in at-risk neonates

*NAMPT* expression in whole blood at day 5 of life was analyzed in 36 preterm neonates matched for very low birth weights (VLBW, birthweight ≤1,500 g) and gestational age (<32 weeks) (Pietrzyk et al., 2013; Moreira et al., 2023). VLBW neonates developing BPD (n = 18) exhibited lower gestational age and birthweights (Table 2) and significantly increased *NAMPT* expression compared to non-BPD neonates (n = 18) (Figure 1H) yielding an area under the receiver operating characteristic curve of 72.3% in predicting BPD at 4 weeks of age (Figure 1I). Importantly, *NAMPT* expression at day 5 predicted severe BPD at 36 weeks of age with an AUC of 72.7%.

### 3.3 eNAMPT-neutralizing mAb reduces neonatal eNAMPT/cytokine levels and improves survival in preclinical murine IAI

**FIGURE 2:**
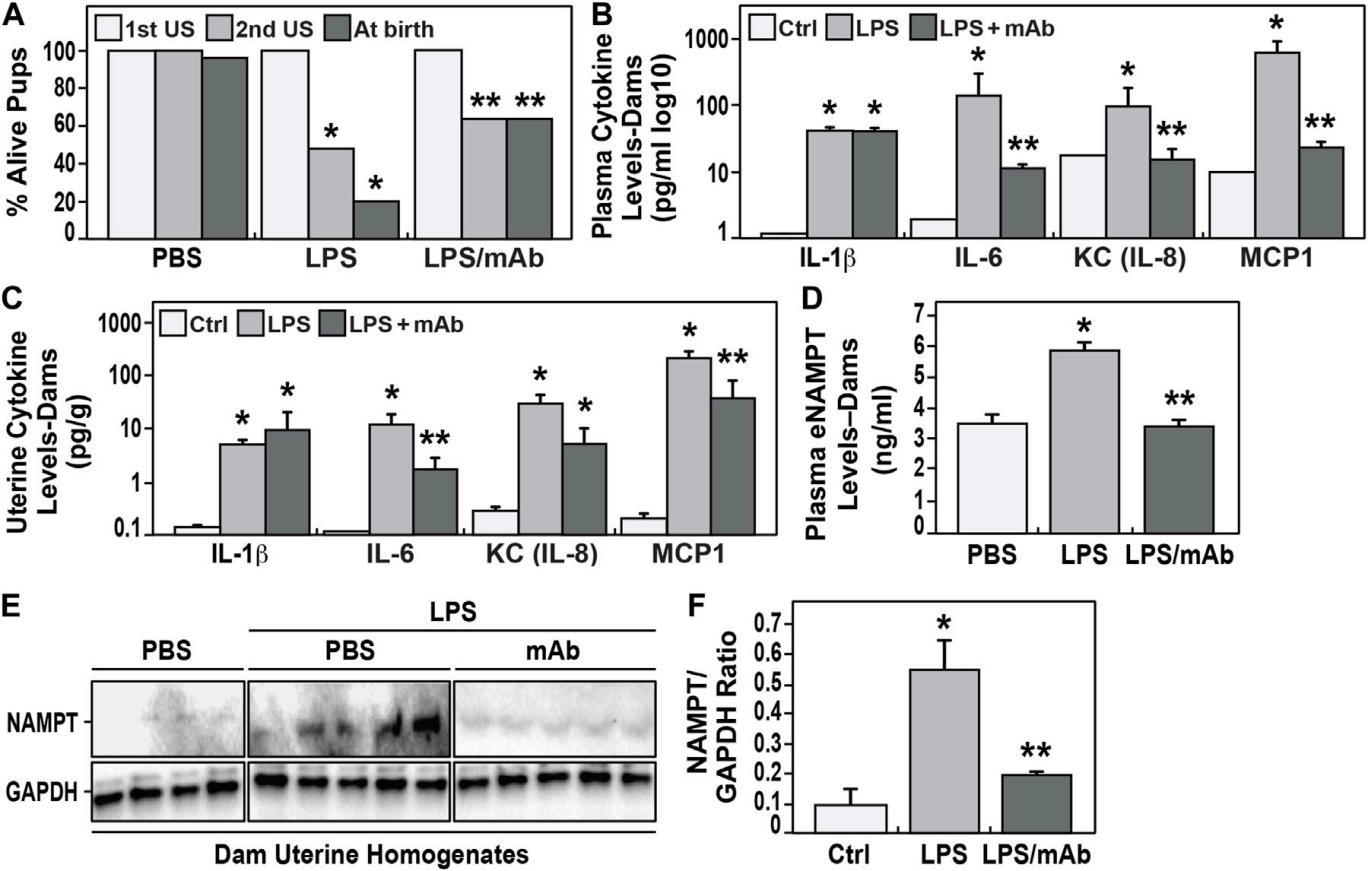
Effect of eNAMPT neutralization on IAI-exposed murine neonate survival and eNAMPT/cytokine levels. **(A)**. Shown are the percentage of alive embryos in pregnant C57Bl6 mice determined at the initial ultrasound (1st US) performed prior to LPS injection and the second ultrasound (2nd US) performed 48 h after LPS injection. Also shown is the percentage of live births among the three studied groups (PBS-exposed, LPS-exposed, LPS-exposed and eNAMPT mAb-treated). The survival rate was significantly reduced in the LPS-exposed group (20%, N = 33) compared to the survival rate in the PBS-exposed group (99%, N = 36). In contrast, the LPS-exposed but eNAMPT-neutralizing mAb-treated group displayed a greater than 3 fold improvement in survival (64%, N = 35) (*p* < 0.05). **(B,C)**. Cytokine levels in maternal plasma and uterine tissue homogenates obtained from pregnant dams after LPS injection. LPS induces significant increases in cytokine expression (IL-1β, IL-6, IL-8, MCP1) in both plasma and uterine tissues with each level attenuated in dams treated with the eNAMPT-neutralizing mAb with the exception of IL-1β and IL-8 in uterine homogenates (*p < 0.05 vs. control, **p < 0.05 vs. both control and LPS group). **(D,E,F)**. eNAMPT expression level in maternal plasma and uterine tissue homogenates obtained from pregnant dams after LPS injection. LPS induces significant increases in eNAMPT expression in plasma **(D)** and uterine tissues **(E,F)** which is attenuated in the mice receiving the eNAMPT-neutralizing mAb (*p* < 0.05). Western blot intensities were quantified by densitometry measurements with data represented as mean ± SE. *p < 0.5.

The first dam ultrasound examination at G14 (prior to LPS injection) demonstrated ∼100% fetal viability which dramatically fell to ∼42% viability after LPS exposure (second ultrasound, 24–36 h after LPS) (Figure 2A). Pup survival at birth from LPS-challenged dams was reduced to 20% in association with the rapid onset of preterm birth (day 18) compared to unchallenged dams with 100% survival and full term birth at day 21. Measurements of blood cytokines in LPS-exposed dams at 24 h demonstrated systemic inflammation with highly significant elevations in IL-1β, IL-6, KC, MCP-1 (Figure 2B) and eNAMPT levels (Figure 2D) when compared to unexposed dams.

In contrast, LPS-challenged dams receiving the eNAMPT-neutralizing mAb exhibited significantly higher fetal viability at 2nd ultrasound (64% vs. 42%), and significant reduction in preterm births (100% day 21 births), and significantly greater birth survival (>60%) (Figure 2A). These results were strongly supported by highly significant reductions in plasma cytokine levels (Figure 2B) and plasma eNAMPT levels (Figure 2D) as well as significant reduction in cytokines (except IL-1β) and eNAMPT protein expression in uterine tissue homogenates collected from LPS-exposed dams compared to controls (Figures 2C,E,F).

### 3.4 eNAMPT neutralization reduces cytokine burden and risk of BPD development in hyperoxia-exposed murine neonates

**FIGURE 3:**
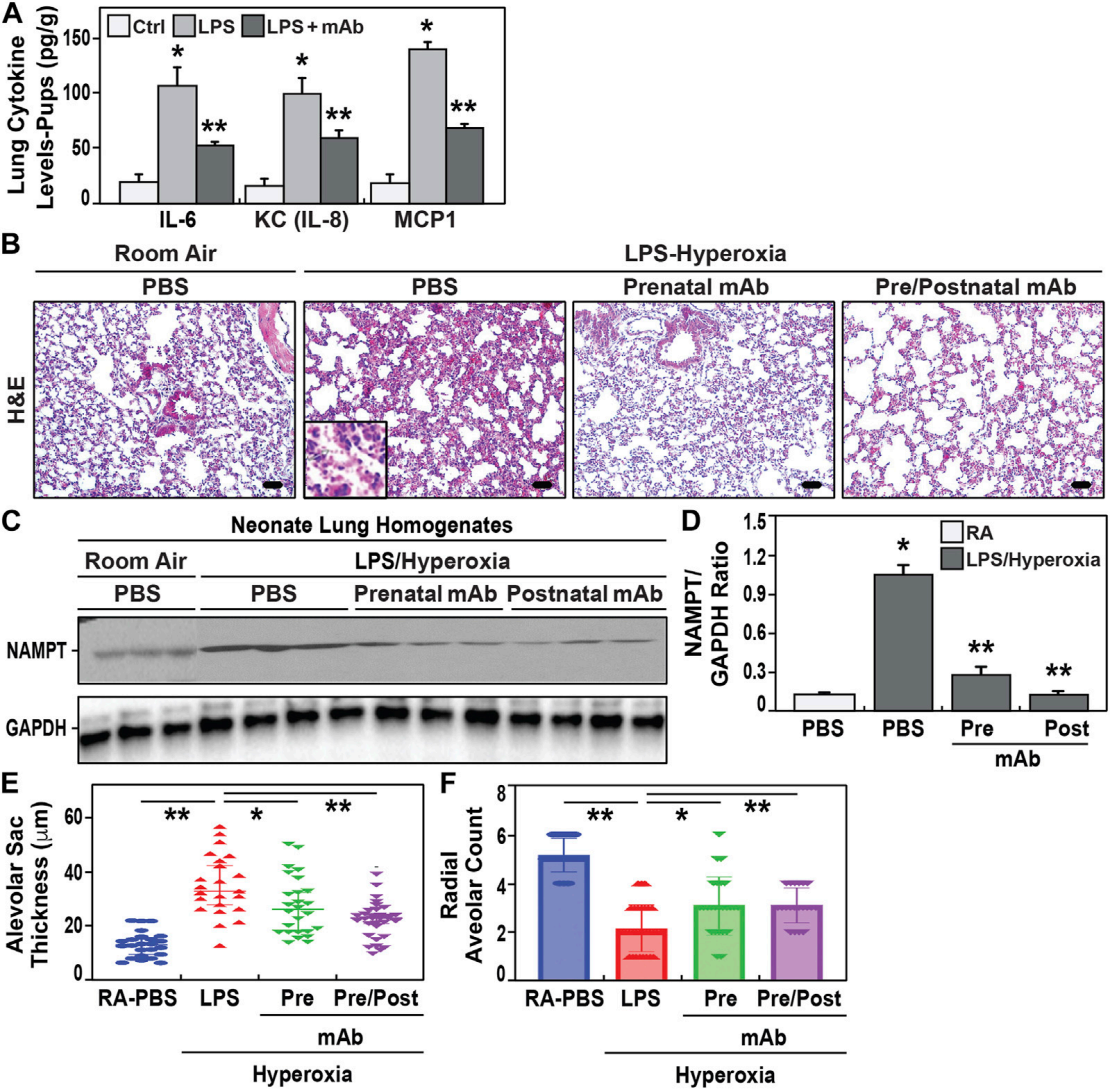
Effect of eNAMPT mAb on development of BPD in hyperoxia-exposed neonates. **(A)**. Cytokine levels (IL-6, IL-8, MCP1) were determined in neonatal lung tissue homogenates at 24h of life. Neonates born to LPS- challenged dams showed significant increased cytokine expression compared to PBS- challenged dams. These increases were attenuated in neonates born to LPS-challenged dams treated with the eNAMPT mAb prenatally (mean ± SE, N = 10 animal/group, *p < 0.05 vs. control, **p < 0.05 vs. both control and LPS group). **(B)**. H&E staining of lung sections at 21 days of age from neonates exposed to either room air or to 2 weeks of hyperoxia (“two hit model”). Neonates born to LPS-challenged dams showed greater inflammatory injury with leukocyte infiltrate, disruption of alveolar septa and interstitial thickening compared to neonates born to LPS- challenged dams treated prenatally with eNAMPT mAb. Scale bar = 50 μm. **(C,D)**. Densitometric summary of Western blot intensities of eNAMPT protein expression in neonatal lung homogenates at 21 days of age with data represented as mean ± SE. *p < 0.5. Neonates born to LPS-challenged dams that were exposed to hyperoxia showed significantly enhanced NAMPT expression. Similarly exposed neonates receiving the eNAMPT-neutralizing mAb prenatally only or pre and postnatal administered showed reduced NAMPT expression (N = 5 animal/group). **(E,F)**. Morphometrics studies were performed on neonate lung sections obtained at 21 days of age. Significant increases in alveolar sac thickness and a significant decreases in radial alveolar counts were observed among hyperoxia-exposed neonates born to LPS-challenged dams compared to hyperoxia-exposed neonates born to dams treated prenatally with mAb or dams who received both prenatal and postnatal mAb (*p* < 0.05, mean ± SE, n = 10 animals/group).

We next observed neonatal pups born to IAI-challenged dam to exhibit significant increases in lung tissue expression of IL-6, KC, and MCP-1 when compared to pups from control dams (Figure 3A, 24 h postnatal). In contrast, pups born to LPS-challenged dams receiving the eNAMPT-neutralizing mAb displayed significant reductions in each cytokine assayed (∼50% reduction) (Figure 3A). Next, surviving pups born to IAI-challenged dams were postnatally-exposed to 85% oxygen (14 days). This resulted in histologic evidence of severe lung inflammation (Figure 3B), marked NAMPT expression in lung tissues (Figures 3C,D), and significant increased development BPD reflected by increased alveolar sac thickness (Figure 3E) and reduced radial alveolar septation (Figure 3F) (compared to room air-exposed pups). In contrast, pups born to IAI-challenged dams receiving eNAMPT-neutralizing mAb, either prenatally alone or both prenatally and postnatally, exhibited significant attenuation of histologic lung inflammation (Figure 3B), NAMPT tissue expression (Figure 3D) and significant reductions in lung morphogenesis indices of BPD (Figures 3E,F).

### 3.5 eNAMPT neutralization reduces pulmonary hypertension (PH) risk/severity in hyperoxia-exposed neonates

**FIGURE 4:**
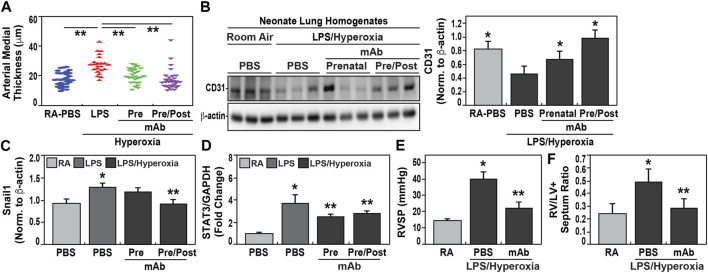
Effect of eNAMPT mAb on development of pulmonary hypertension in hyperoxia-exposed neonates. **(A)**. Measurements of arterial wall thickness was significantly increased among hyperoxia-exposed neonates born to LPS-challenged dams compared to room air-exposed neonates from PBS-challenged dams. In contrast, hyperoxia-exposed neonates born to LPS-challenged dams treated prenatally with eNAMPT mAb, either solely prenatally or both prenatally and postnatally, demonstrated highly significant reductions in arterial wall thickness approaching values of control neonates. (**p < 0.01). **(B)**. CD31 immunoreactivity, a marker of angiogenesis, was markedly reduced in Day 21 lung homogenates obtained from hyperoxia-exposed neonates born to LPS-challenged dams compared to room air-exposed neonates from PBS-challenged dams. In contrast, CD31 expression was restored in hyperoxia-exposed neonates born to LPS-challenged dams treated prenatally with mAb either prenatally or prenatally and postnatally with restoration significantly superior in neonates receiving both mAb doses. This was captured by densitometric quantification of western blots, normalized to β-actin (*p* < 0.05). **(C,D)**. RT-PCR-based measurements of Snail1 and STAT3 mRNA expression was performed utilizing isolated neonatal lung RNA. Both Snail1 and STAT3 mRNA expression were significantly elevated among hyperoxia-exposed neonates born to LPS-challenged dams compared to room air-exposed neonates from PBS-challenged dams. These increases in RNA expression were significantly blunted in hyperoxia-exposed neonates born to dams treated prenatally with mAb only or pre and postnatally (*p < 0.05 vs. control, **p < 0.05 vs. both control and LPS group). **(E,F)**. Hemodynamic studies performed at day 21 of neonatal life showed that right ventricular systolic pressure (RVSP) and RV/LV + S ratio were significantly increased in hyperoxia-exposed neonates born to LPS-challenged dams compared to room air-exposed neonates from PBS-challenged dams with highly significant attenuation observed in hyperoxia-exposed neonates born to LPS-challenged dams treated with mAb prenatally (*p* < 0.05). All data are represented as mean ± SE, n = 10 animals/group).

Similar to the development of BPD, we observed increased pulmonary hypertension (PH) in hyperoxia-exposed neonates born to IAI-exposed dams. Significantly increases in vascular remodeling evidenced by increased arterial wall thickness (Figure 4A) and decreased CD31 expression (Figure 4B) when compared to pups born to PBS-exposed controls. Significantly increased mRNA expression of PH markers, SNAIL1 (Figure 4C) and STAT3 (Figure 4D), were observed in neonates born to LPS-exposed dams. These findings were accompanied by significant reductions in both p-SMAD (Supplemental Figure S2A) and eNOS (Supplemental Figure S2B) compared to controls. The development of PH in hyperoxia/IAI-exposed pups was further supported by significant hemodynamic elevations in the right ventricular systolic pressure (RVSP) and Fulton index in neonates born to LPS-challenged dams (Figures 4E,F). Echocardiographic studies demonstrated increased RV fractional wall thickness, pulmonary acceleration time/pulmonary ejection time ratio (PAT/PET), and tricuspid annular plane systolic excursions (TAPSE) (Table 3).

**TABLE 3:** Echocardiographic indices in surviving neonates at day 21.

ECHO data	Control RA	LPS/Hyperoxia + PBS	LPS/Hyperoxia + eNAMPT mAb	*p* Value
Stroke Volume	33.93 ± 4.5	32.57 ± 3.8	34.11 ± 3.9	NS
Cardiac Output/Minute	15.4 ± 1.3	15.2 ± 2.1	14.8 ± 2.2	NS
Ejection Fraction	59 ± 3.1	53 ± 3.3	54+/4.5	NS
RV Fractional Wall Thickening	0.32 ± 0.02	0.44 ± 0.04	0.31 ± 0.01*	0.04
PAT/PET ratio	0.34 ± 0.01	0.25 ± 0.03	0.34 ± 0.01*	0.01
TAPSE	0.669 ± 0.03	0.512 ± 0.03	0.67 ± 0.03*	0.01

(n = 10 mice group). PAT: pulmonary acceleration time; PET: pulmonary ejection time; TAPSE: tricuspid annular plane systolic excursion.

\In contrast, again similar to BPD development, IAI-exposed pups receiving the eNAMPT-neutralizing mAb prenatally, showed attenuation of each index of PH including reduced arterial wall thickness (Figure 4A), preserved CD31 expression (Figure 4B), reduced expression of PH markers, SNAIL1 and STAT3 (Figures 4C,D), and reduced RVSP and Fulton index measurements (Figures 4E,F). The administration of an additional postnatally-delivered eNAMPT mAb dose following hyperoxia exposure resulted in greater protection than prenatal eNAMPT mAb treatment alone with regard to arterial wall thickness (Figure 4A), CD31 and Snail1 expression (Figures 4B,C), and both p-SMAD and eNOS expression (Supplemental Figures S2A,B).

### 3.6 eNAMPT neutralization rectifies IAI-induced gene dysregulation in uterine-maternal and neonatal cardiac tissues

**FIGURE 5:**
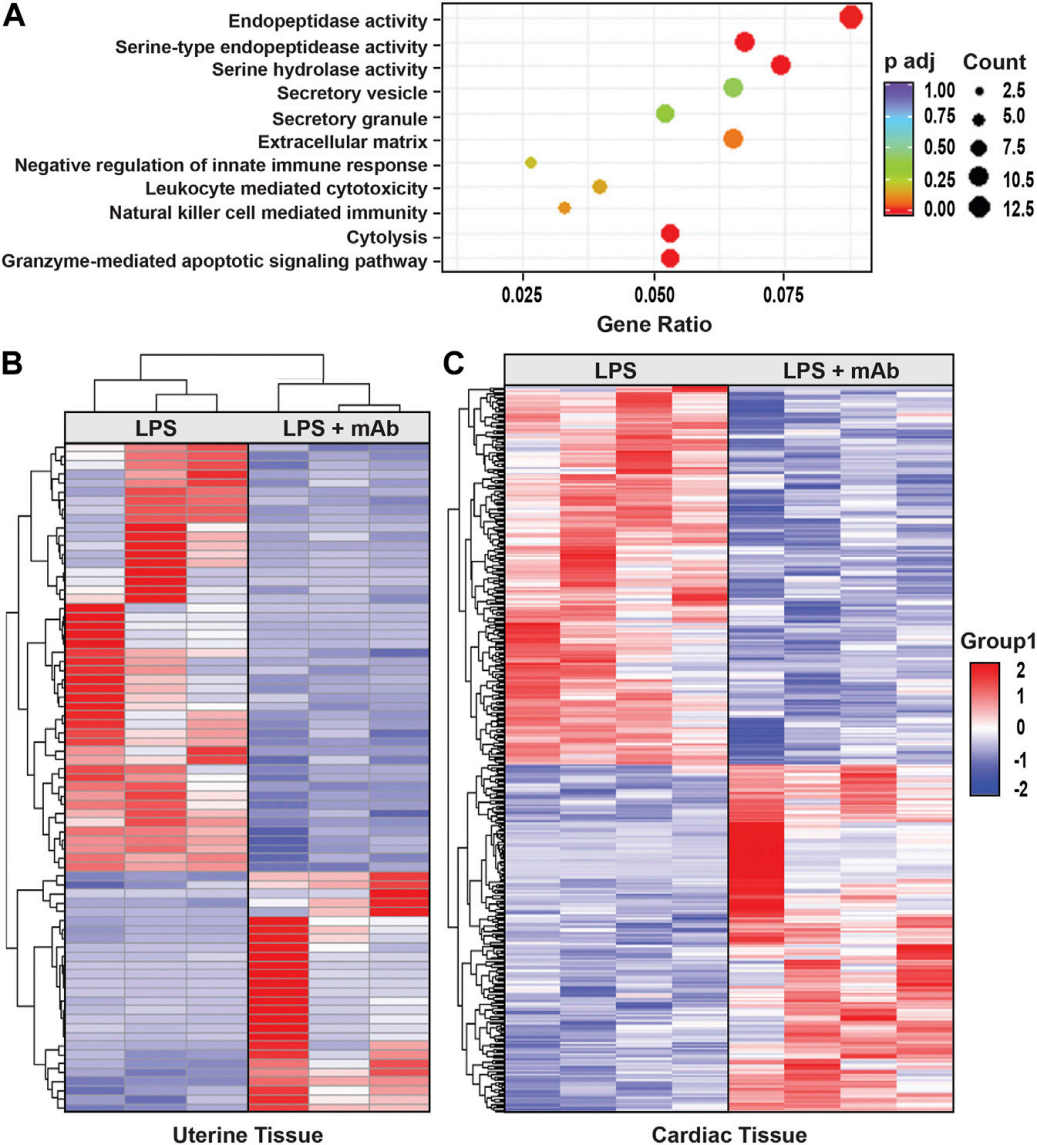
Effect of eNAMPT neutralization on genome-wide gene expression in IAI-exposed dams and surviving neonates. **(A)**. The diameter of the dot is directly correlated to the number of genes that participate in the GO term. The color of the dot indicates the p-adjusted value, and the *X*-axis indicates the proportion of the genes contained in each GO. **(B)**. Shown is the heat map of the 75 DEGs identified in IAI-exposed dams (uterine tissue) receiving the eNAMPT-neutralizing mAb (*p* = 0.01). **(C)**. Shown is the heat map of the 396 cardiac tissue DEGs identified in pups born to IAI-exposed dams receiving PBS compared to dams receiving the eNAMPT-neutralizing mAb (*p* < 0.05, FDR 0.5). Red indicates upregulated genes, blue downregulated genes. Dot Graph showing the Gene Ontology enrichment for biological process, cellular component, and molecular function associated with 177 differentially-expressed genes (DEGs), comparing IAI- and non-IAI-exposed dams.

RNA sequencing and differential gene expression analysis identified tissue-specific differences between IAI- and non-IAI-exposed uterine-maternal tissues and neonatal cardiac tissues. IAI- and non-IAI-exposed uterine tissues yielded 177 differentially-expressed genes (DEGs) (*p* < 0.01, FDR 10%), 83/177 of DEGs were upregulated (FC 1). The top 50 DEGs are presented in Supplemental Table 1 and yielded IAI-related dysregulated pathways associated with innate immune responses, cytotoxicity, inflammation, NK-mediated immunity and autoimmunity (Figure 5A). IAI-exposed dams receiving the eNAMPT-neutralizing mAb revealed 75 uterine tissue DEGs (*p* value < 0.01, Table 4) related to MAP kinase signaling, surfactant metabolism, and Wnt signaling. Finally, we analyzed the effect of the eNAMPT-neutralizing mAb on cardiac tissue gene expression in surviving neonates from IAI-exposed dams identifying 396 DEGs (*p* < 0.05, FDR = 0.5, Table 5) related to cysteine/methionine metabolism, lipolysis in adipocytes, and RAGE signaling. Visualization of DEGs in both dams and surviving neonates following eNAMPT-neutralizing mAb are shown in Figures 5B,C. Together, these genomic results are consistent with involvement of eNAMPT/TLR4 signaling-influenced genes in IAI pathobiology, severity and mortality.

**TABLE 4:** Kegg Pathway Analysis in Uterine Tissues from LPS-Challenged Dams with PBS vs. eNAMPT mAb Treatment.

Source	Description	*p* value	*p* value adj	Genes
Reactome	Surfactant metabolism	0.002	0.046	*Lmcd1, Adora2B*
Reactome	RAF/MAP kinase cascade	0.003	0.046	*Rasal3, GfrA1, Fgf1, Camk2b*
Reactome	MAPK1/MAPK3 signaling	0.003	0.046	*Rasal3, GfrA1, Fgf1, Camk2b*
Reactome	MAPK family signaling cascades	0.004	0.049	*Rasal3, GfrA1, Fgf1, Camk2b*
Reactome	G alpha (z) signaling events	0.005	0.049	*RGS16; ADRA2C*
KEGG	Wnt signaling pathway	0.008	0.069	*Wnt11,Camk2b, Sfrp4*

**TABLE 5:** KEGG Pathway Analysis of Cardiac DEGs from LPS-Exposed Dams +/- eNAMPT mAb.

Description	*p*-value	*p*-value adj	Gene name
Cysteine and methionine metabolism	1.02E-02	8.31E-01	*Mtr/Amd2/Mpst/Tst/Mat2a*
Fatty acid degradation	1.17E-02	8.31E-01	*Gm16984/Cpt2/Acadl/Gcdh*
Sulfur metabolism	1.53E-02	8.31E-01	*Mpst/Tst*
Regulation of lipolysis in adipocytes	1.95E-02	8.31E-01	*Aqp7/Pnpla2/Akt2-ps/Nppa*
Endocrine resistance	2.33E-02	8.31E-01	*Jun/Mmp2/Notch3/Akt2-ps/Erbb2*
Biosynthesis of unsaturated fatty acids	2.45E-02	8.31E-01	*Scd4/Acot7/Acot1*
Proteoglycans in cancer	3.15E-02	8.31E-01	*Lum/Mmp2/Gpc1/Dcn/Fzd2/Akt2-ps/Wnt2/Erbb2*
AGE-RAGE signaling pathway in diabetic complications	3.67E-02	8.31E-01	*Jun/Mmp2/Akt2-ps/Plcd3/Col3a1*
Breast cancer	3.90E-02	8.31E-01	*Jun/Notch3/Fzd2/Akt2-ps/Wnt2/Erbb2*

## 4 Discussion

We addressed the serious unmet need for therapies that reduce preterm births and adverse fetal outcomes by targeting eNAMPT, a critical innate immunity DAMP and TLR4 ligand. Supporting the focus on eNAMPT as an IAI/IUI target was the observation that women with chorioamnionitis showed dramatic placental NAMPT expression (Figure 1), compared to non-chorioamnionitis women. Blood eNAMPT levels (Figure 1) were elevated in both chorioamnionitis and non-chorioamnionitis cases but were not significantly different, a finding similar to previous reports (Mazaki-Tovi et al., 2009; Pavlova et al., 2018; Musilova et al., 2021). Prior analyses of amniotic fluid cell-free RNA transcriptomes from women with preterm births demonstrated prominent *NAMPT* dysregulation (Bhatti et al., 2021) with eNAMPT levels in amniotic fluids reflecting the presence of IAI/chorioamnionitis (Mazaki-Tovi et al., 2009; Giomisi et al., 2011; Pavlova et al., 2018; Musilova et al., 2021). Thus, while serum eNAMPT levels may not serve as a useful biomarker of chorioamnionitis, eNAMPT levels in amniotic fluids may potentially serve as a future marker of IAI/chorioamnionitis.

Importantly, our studies of very low birthweight (VLBW) preterm infants revealed that elevated whole blood *NAMPT* expression on day 5 to significantly predict BPD development suggesting eNAMPT protein/gene expression may serve as a novel biomarker for early prediction of chronic lung disease among VLBW neonates. Additional biomarkers of chorioamnionitis/BPD include serum Krebs von den Lungen (KL-6), a glycoprotein preferentially expressed and secreted by type II pneumocytes and bronchial epithelial cells. KL-6 expression has been correlated with early prediction of BPD (Ogihara et al., 2006). Clara cell secretory protein (CC16), a protein secreted by the tracheobronchial epithelium, is another marker for lung epithelial injury whose elevated serum levels at 72 h after birth were highly predictive of BPD development (Sarafidis et al., 2008). Finally, elevated levels of neutrophil gelatinase-associated lipocalin (NGAL) at birth, a glycoprotein expressed by granulocytes, is a predictor of BPD among neonates with GA < 31 weeks (Inoue et al., 2013).

Our human studies implicating eNAMPT as a target in IAI and preterm births, were strongly supported by preclinical studies in a murine IAI model of preterm births that showed an eNAMPT-neutralizing mAb to significantly improve pup survival, reduce neonate lung inflammation, and reduce the severity of hyperoxia-induced BPD and PH. These results underscore the eNAMPT/TLR4 inflammatory pathway as a highly druggable contributor to IAI pathobiology during pregnancy. Currently, the efficacy of anti-inflammatory therapeutics to prevent preterm births and reduce adverse outcomes, including anti-TNFα (Lamont, 2003; Winger and Reed, 2008; Renaud et al., 2011; Gelber et al., 2015) and anti-IL-1β strategies (Romero et al., 1991; Sadowsky et al., 2000; Hirsch et al., 2006; Sadowsky et al., 2006; Kallapur et al., 2009; Girard et al., 2010; Nadeau-Vallee et al., 2015; Nadeau-Vallee et al., 2016; Rueda et al., 2016; Nadeau-Vallee et al., 2017; Presicce et al., 2018; Lei et al., 2019), have been inconclusive (Lockwood, 2002; Hirsch et al., 2006; Leitner et al., 2014) and well-controlled human studies are lacking. Prior studies suggesting TLR4 involvement in IAI-induced preterm delivery emphasized microbial-mediated activation of IAI responses *via* TLR4 function as a pathogen recognition receptor (Li et al., 2010). In contrast, our studies underscore the role of the novel DAMP and TLR4 ligand, eNAMPT, and the eNAMPT/TLR4 pathway as a druggable inflammatory cascade that contributes to human and murine IAI pathobiology in pregnancy. This supported by multiple preclinical studies of organ inflammation involving the eNAMPT-neutralizing mAb which consistently ameliorated NFkB phosphorylation, reflecting dampened TLR4 activation of innate immunity (Ahmed et al., 2021; Garcia et al., 2022a; Garcia et al., 2022b; Sammani et al., 2022; Sun et al., 2023; Tumurkhuu et al., 2023).

Our studies specifically assessed the capacity of the eNAMPT mAb to both improve pup survival, and mitigate the propensity for surviving neonates to develop BPD and PH (Thome et al., 1998; Choi et al., 2006). Mechanistically, we speculate the eNAMPT/TLR4-neutralizing mAb to inhibit intrauterine inflammation and triggering of the FIRS process responsible for increasing risk for preterm postnatal morbidities and mortalities. Although preliminary, our studies suggest that a second mAb dose postnatally delivered directly to the newborn pup may act as a booster to attenuate the deleterious effects of the postnatal oxidative stress challenge induced by hyperoxia exposure. The targeting of eNAMPT (Jensen and Schmidt, 2014; Kalikkot Thekkeveedu et al., 2017) and the TLR4 inflammatory cascade in BPD (Lavoie et al., 2012) has been suggested in the disrupted alveolar and pulmonary vascular growth characteristic of BPD although whether maternal IAI increases BPD risk remains controversial (Bose et al., 2009; Hartling et al., 2012; Jobe, 2012). Further, hyperoxia-induced BPD leads to upregulation of the proinflammatory miRNA, miR-34a (Savani, 2018; Das et al., 2021), that regulates *NAMPT* transcription (Pi et al., 2021). Unlike other miRs (Adyshev et al., 2014), the effect of miR-34a on eNAMPT secretion and eNAMPT/TLR4-induced lung injury is unclear (Adyshev et al., 2014). The important finding that *NAMPT* expression significantly predicted BPD development in VLBW infants suggests blood eNAMPT protein/mRNA levels may identify at-risk neonates during the first week of life.

PH is another chronic complication of preterm births occurring in ∼25% of preterm infants (Watterberg et al., 1996; Hansen et al., 2010; Bhat et al., 2012; Eriksson et al., 2015; Mourani et al., 2015; McLaughlin and Gardener, 2016). The targeting of eNAMPT/TLR4 to mitigate the development of PH is a logical extension of our prior work (Chen et al., 2017; Sun X. et al., 2020), including our recent report demonstrating the capacity of the eNAMPT-neutralizing mAb to halt and reverse PH severity in an adult hypoxia/Sugen exposed rat model of PH (Ahmed et al., 2021). PH is especially prevalent in infants with severe BPD with postnatal ventilator-induced injury (Taglauer et al., 2018) and hyperoxia exposure contributing to PH development (Yee et al., 2011; Chao et al., 2018). In our studies, treatment with the eNAMPT Ab improved multiple PH indices and nearly normalized RVP measurements (Figure 4) strongly supporting eNAMPT neutralization as a strategy to significantly improve both short term outcomes (survival at birth) and development of chronic lung disease.

A strength of our studies, in addition to the utilization of both human and murine preclinical samples to validate eNAMPT as a highly novel IAI target, is the integration of histologic, biochemical and genomic approaches in a preclinical murine IAI pregnancy model. For example, genomic studies confirmed strong dysregulation of inflammatory and apoptotic pathways in IAI-exposed dam uterine tissues and cardiac tissues from surviving neonates which were rectified by the eNAMPT-neutralizing mAb. Our studies also exhibited important limitations, however, including the use of an LPS-induced IAI model rather than live bacteria-induced IAI. The use of LPS mirrors only gram-negative bacteria whereas chorioamnionitis is also induced by gram-positive bacteria, fungi, virus, parasites, and *mycoplasma*. Another important study limitation is the absence of genomic analysis of murine neonatal lung tissues, an undoubtedly highly informative approach to understand the effect of the eNAMPT-neutralizing mAb on gene dysregulation induced by the “2-hit” model of IAI and hyperoxia in the context of BPD and PH (Velten et al., 2010).

In conclusion, our human and preclinical IAI studies are highly consistent with the central involvement of the eNAMPT/TLR4 inflammatory cascade in IAI pathobiology with eNAMPT/TLR4 pathway identified as a highly druggable IAI target. Although preterm births are a continued global healthcare challenge, these exciting results demonstrated strong attenuation of IAI severity by the eNAMPT-neutralizing mAb with delayed preterm birth and significantly improved neonatal outcomes thereby supporting future clinical trials designed to test the eNAMPT-neutralizing mAb as a therapeutic strategy to impact women with IAI at risk for adverse fetal outcomes, a major global unmet need.

## Data Availability

The datasets presented in this study can be found in online repositories. The names of the repository/repositories and accession number(s) can be found below: BioProject ID PRJNA923324.
