# Supplementary figures and images for: The eNAMPT/TLR4 inflammatory cascade drives the severity of intra-amniotic inflammation in pregnancy and predicts infant outcomes
Source: Front Physiol. 2023 Jun 20;14:1129413. doi: 10.3389/fphys.2023.1129413 (PMC10319582; doi:10.3389/fphys.2023.1129413)

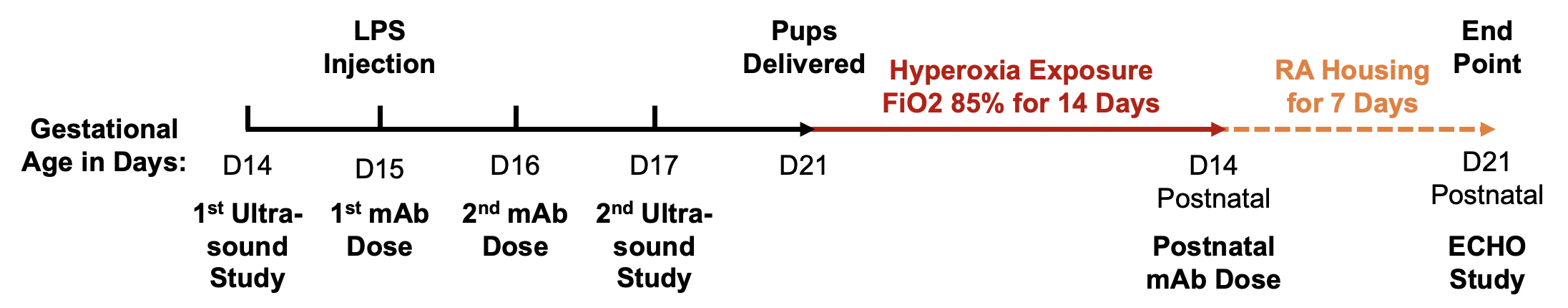

Supplement: Supplementary file 1 [file Image1.JPEG]

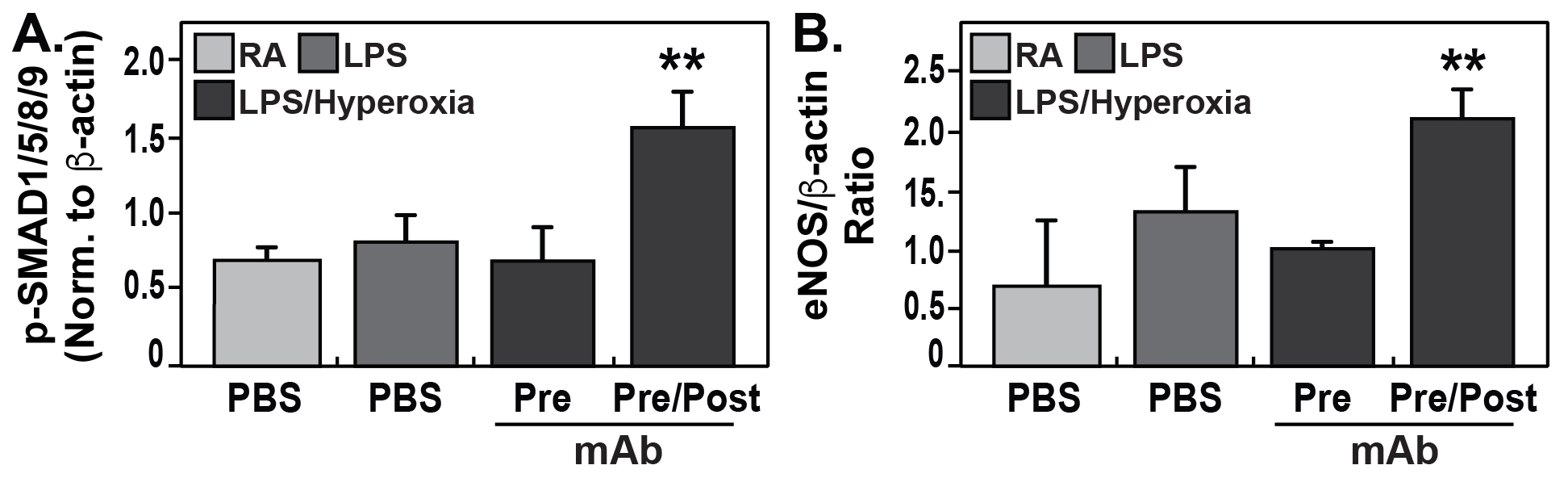

Supplement: Supplementary file 2 [file Image2.JPEG]
