# Supplementary material for: The eNAMPT/TLR4 inflammatory cascade drives the severity of intra-amniotic inflammation in pregnancy and predicts infant outcomes
Source: Front Physiol. 2023 Jun 20;14:1129413. doi: 10.3389/fphys.2023.1129413 (PMC10319582; doi:10.3389/fphys.2023.1129413)
Supplement: Supplementary file 3 [file DataSheet1.pdf]

Supplementary Methods

Human pregnant subject cohorts. Demographic data of the pregnancy groups with and without clinical chorioamnionitis are shown in **Table 1.** The key clinical findings associated with clinical chorioamnionitis include fever, uterine fundal tenderness, maternal tachycardia (>100/min), fetal tachycardia (>160/min) and purulent or foul amniotic fluid, versus non-chorioamnionitis pregnant control group (4). Pregnant mothers with clinical diagnosis who were admitted to the hospital with clinical diagnosis of chorioamnionitis were included. After consenting, blood samples were collected and assayed for eNAMPT serum levels. After birth, all placentas and chorions were sent for pathological examination. The chorioamnionitis diagnosis was confirmed after birth based on pathologic analyses based on the definition recommended by the Amniotic Fluid Infection Nosology Committee of Perinatal Section, the Society for Pediatric Pathology (42). The first reliable histological indicator of a maternal inflammatory response to infection is a diffuse neutrophil band either at the chorion or at the subchorionic space (stage 1). Later, neutrophils cross the chorion to enter the chorionic connective tissue and/or amnion (stage 2). Apoptosis and neutrophil fragmentation are among the defining features of stage 3 of the maternal inflammatory response, called necrotizing chorioamnionitis." Pathologically studied cases showed abnormal findings and classified as grade1 and grade1/2. These cases were labeled as "chorioamnionitis" cases (N=40) Cases without pathological evidence of chorioamnionitis was labeled as "without chorioamnionitis" (N=27). Finally, blood was collected from a third group of control healthy women and assayed for serum eNAMPT levels (N=6).

Human neonatal subject cohorts. To evaluate the impact of NAMPT expression on the adverse events occurring in VLBW neonates, a secondary data analysis of peripheral blood microarray dataset (GSE32472) from the National Library of Medicine's Gene Expression Omnibus (https://www.ncbi.nlm.nih.gov/geo/) was performed. Details pertaining to the GSE32472 dataset have been previously reported (51) involving 97 preterm newborns with a gestational age <32 weeks, birthweight ≤1500 grams, who required respiratory support at the time of enrollment, and survived hospitalization. Blood whole microarray gene expression on day 5 was examined evaluate *NAMPT* expression as an early predictor of bronchopulmonary dysplasia (BPD), defined per National Institute of Child Health and Human Development guidelines as infants requiring supplemental oxygen at 28 days of age (52). Secondary analysis included comparison of groups without BPD to neonates with severe BPD defined as positive pressure ventilation or oxygen support ≥30% at 36 weeks postmenstrual age (52). Institutional review board approval was not required as this study used publicly-available de-identified information.

Reagents. Unless otherwise noted, all reagents were purchased from Sigma-Aldrich (St. Louis, MO). Antibodies immunoreactive against β-actin from Invitrogen (Carlsbad, CA). Goat, rabbit, and mouse secondary antibodies were purchased from Life Technologies (Waltham, MA). Human PBEF/Visfatin Biotinylated Antibody (cat #BAF4335) was purchased from R&D Systems; Ultra Streptavidin-HRP (cat #N504) from ThermoFisher Scientific; Actin-HRP (cat #A3854-200UL) from Sigma-Aldrich. Peroxidase AffiniPure Goat Anti-Mouse IgG (H+L) (cat# 102646-170) and Anti-IgG (H+L) Goat Polyclonal Antibody (Horseradish Peroxidase) (cat #102645-188) and IgG for use as controls were obtained from Jackson ImmuneResearch (West Grove, PA). The eNAMPT-neutralizing humanized mAb, ALT-100, was provided by Aqualung

Therapeutics Corporation (Tucson, AZ) and was generated from murine eNAMPT-neutralizing mAbs as we have previously described (33, 40, 41).

Immunohistochemistry (IHC) for eNAMPT in human placentas. Chorion tissues were collected after birth from pregnant women with and without IUI, fixed in 10% Neutral Buffered formalin for a minimum of 48 h, embedded in paraffin, sectioned, and mounted onto slides. IHC staining for NAMPT (5 um sections) was performed using the avidin-biotin-peroxidase method to visualize lung tissue expression of NAMPT with anti-NAMPT (Bethyl Laboratories, Montgomery, TX) or with a matched protein concentration (1ug/ml) of rabbit IgG (Vector Labs, Burlingame CA) as we have previously reported (33, 34, 40).

Immunostaining analysis. Digital images obtained from Confocal software were exported to ImageJ. The fluorescence intensity associated with each pixel was determined in sections 750 × 750 μm that included 4 sections per animal and 5 animals per group. Excitation and acquisition parameters were adjusted to fully eliminate pixel saturation, and all images were collected under identical settings.

Murine model of intrauterine inflammation. All mice were housed under standard conditions (12h light-dark cycle, 25-27°C, ~40% humidity) in autoclaved micro-isolator cages with free access to food and water throughout the duration of the experiments. All animal care procedures and experiments were approved by the Institutional Animal Care and Use Committee (University of Arizona).

Timed-pregnant C57BL6 mice (aged 8-10 weeks) with the vaginal plug were documented as day 0 of gestation and confirmed by serial physical examinations and abdominal ultrasound. LPS (*E. Coli* , 055:B5, Sigma, 50 ug/mouse) was administered IP to pregnant mice at day 15 of gestation to produce high-grade placental inflammation (43), intrauterine inflammation, maternal systemic inflammatory response, a well-established model of clinical intrauterine inflammation (44, 45) that results in severe IAI 80-90% of dams and moderate to high rates of fetal loss (46) with premature abortion within 24-48 hrs (44, 45, 47). Treated dams group received two doses of eNAMPT mAb (10ug/mouse IP) on day GD15 and GD16 S9upp. Fig. 1).

Ultrasound evaluation of fetal viability. The initial abdominal ultrasound was performed prior to LPS challenge to define the number of sacs in each horn of each dam. The second abdominal ultrasound was performed 48 hr after LPS administration and was designed to determine the number of total fetuses (dead or alive) in each dam. After birth, the number of surviving pups in each group was recorded to determine the survival rate.

Biomarker measurements. A Measurements of serum, plasma and lung/uterine homogenate biomarker levels of IL-6, KC/IL-8, IL-1β, TNFα, and eNAMPT were measured utilizing meso-scale ELISA platform (Meso Scale Diagnostics, Rockville, MD) as we have previously described (31, 44, 48).

Western blotting for eNAMPT protein expression. Snap-frozen lung tissues were homogenized in RIPA buffer (50 mmol/L Tris-HCl pH 7.4, 150 mmol/L NaCl, 0.5 % sodium deoxycholate, 0.1 % SDS, 1% NP-40, 5 mmol/L EDTA) supplemented with complete protease/phosphatase inhibitor cocktail (Cell Signaling Cat #5872S) using tissue grinder with

glass pestles (VWR Cat #26307-606). After centrifugation (15,000 g for 20 min at 4°C), protein concentration of homogenates was determined by Bio-Rad DC protein assay (cat #5000112). Following incubation 5 min at 90°C in loading buffer, aliquots containing equal amounts of protein (25–30 ug) were subjected to sodium dodecyl sulfate polyacrylamide gel electrophoresis (SDS-PAGE). Subsequently, proteins were transferred to PVDF membranes and probed with specific primary, then by secondary antibodies. Proteins were visualized using an ECL system (Pierce West Pico cat #34580) and ChemiDoc MP imaging system (Bio-Rad). Densitometric analysis was performed using Bio-Rad Image Lab 6.01.

Assessment of murine BPD. As shown before (48), two hit model, with prenatal inflammation and postnatal hyperoxia produced a greater degree of lung injury, with significantly enlarged alveoli, pulmonary fibrosis, and macrophage infiltrate, than either a prenatal or postnatal insult alone. In the model of intraamniotic inflammation described above, surviving pups were exposed to hyperoxia FiO2 85% for 14 days, then housed on room air. Exposed groups were studied for long term outcome. To avoid O_2 toxicity in the dams and to eliminate maternal effects between the groups, the nursing dams were rotated between their hyperoxic and room air litters every 24 h. In a subgroup of pups born to treated dams, an additional eNAMPT mAb dose was administered IP after hyperoxia exposure on day 14-15 of life (one dose of eNAMPT mAB, 10ug/mouse IP).

Assessment of murine pulmonary hypertension. Neonatal lungs were collected after 3 weeks of age for lung tissue morphology, fixed in 10% Neutral Buffered formalin, embedded in paraffin, sectioned, mounted onto slides, and finally stained with hematoxylin-eosin (H&E) (40). Neonatal lungs were also examined by western blotting for eNAMPT expression as an index of inflammatory process, for CD31 as an index of angiogenesis, and for SNAIL1, as an index of PH. RT-PCR was also performed for p-SMA (2, 88-90); eNOS and STAT3 as marker of PH**.**

Hemodynamic measurements were evaluated 1-2 weeks post hyperoxia exposure as we have described (49) utilizing a Millar catheter inserted in right jugular vein and into the right ventricle. Right ventricular systolic pressure (RVSP) was measured and recorded using a computerized hemodynamic recording system (HAEMODYN, Harvard Apparatus, MA, USA). RV/S + LV ratio was expressed as a ratio of the weight of the right ventricle to that of the septum plus left ventricle (RV/S + LV), (30, 50).

Quantification of micro-vessel density and vascular wall thickness. The left lung was perfused with 4% paraformaldehyde (PFA), inflated by infusion of 4% PFA at a constant pressure of 25 cm H_2O through a cannula inserted in the trachea, fixed in 4% PFA overnight at 4°C and then embedded in OCT, Subsequently 5-μm-thick sections were taken and stained with hematoxylin and eosin (H&E) Images of individual pulmonary arteries were captured using a digital camera, mounted on a light microscope, and linked to a computer. Microvessel density was quantified by counting the percentage area of positive pixels per image with at least 21 images per sample (5 animals, 3 samples per animal, and 7 sections per sample as described previously) (49).

Vascular wall thickness. Vascular wall thickness was measured using a Zeiss Axiovert 200M light microscope – CCD camera AxioCam (mRm) color camera and expressed as the percentage of total vessel size. Percent wall thickness was calculated as (2 x wall thickness)/external

diameter. External diameter and internal diameter of 50 alveolar vessels (with an external diameter of 100–200 μm) per animal were determined and recorded by an independent investigator blinded to the treatment regimen. The ratio of vessel wall area to total area (WA%) and the ratio of pulmonary arteriole wall thickness to vascular external diameter (WT%) were measured using Zeiss axial program of 3 random wall sections.

Echocardiographic studies. Echocardiography was performed using a Vevo 3100 High Resolution Imaging System (Visual-Sonics, Toronto, Canada) with an MX550D (mouse) designed for rodent cardiac imaging as we have reported (33). Following anesthetic induction in 3% isoflurane, animals were placed in a supine position on a heated platform to maintain body temperature of 37°C. Anesthesia was maintained with 1.5-3% isoflurane (USP, Phoenix) in 100% oxygen and echo images collected and stored as digital cine loops for off-line calculations. Standard imaging planes, M-mode, Doppler, and functional calculations were obtained according to American Society of Echocardiography guidelines and as described in our previous studies (33).

Whole microarray data analysis in VLBW neonates. From a total of 33,252 genes per patient a total of 20,697 genes were included for analysis after filtering out genes with expression levels <50% of total expression. Outliers were weighted according to Ritchie et al (91) and genes were considered significant if the false discovery rates (FDRs), by Benjamini and Hochberg-adjusted P values, were less than 5%. Two *NAMPT* gene probes meeting the FDR threshold when comparing no BPD to BPD or severe BPD were included in the microarray data.

RNA sequencing of murine tissue samples. Mouse uterine and heart RNA was extracted and RNA QC assessed by RIN value, 28S/18S and fragment length distribution (Aligient 2100 Bio analyzer, Agilent RNA 6000 Nano Kit). Following library construct, RNA was sequenced using Illumina Hiseq (NovaSeq) PE150 platform; averagely generating 6 Gb raw data per sample. RNaseq data bioinformatic analyses pipeline included data quality control, calculation of Pearson correlations of all genes expressed to reflect the correlation of gene expression between samples. The HISAT2 and (Hierarchical Indexing for Spliced Alignments of Transcripts) Bowtie2 programs were utilized to align and clean reads to the reference genome and to the reference genes (12, 92, 93). Abundance and distribution of transcripts were assessed obtaining expected number of Fragments Per Kilobase er Millions base pairs (FPKM) (94). Correlation analysis to asses variation between samples was performed by Pearson correlation. DEseq2 algorithms were used to detect DEGs with Bioconductor software packages (95). To control for multiple testing error, the adjusted P-value False Discovery Rate (FDR) was utilized (54). Enrichment analysis for Gene ontology (GO) classification was performed focused on biological process and pathway classification with KEGG and Reactome sources (55).

Statistical analysis. Continuous data were compared using nonparametric methods and categorical data by chi square test. Where applicable, standard one-way ANOVA was used and groups were compared using the Newman-Keuls test. Differences between groups were considered statistically significant when p values were less than 0.05 ($p < 0.05$). Two-way ANOVA was used to compare the means of data from two or more different experimental groups. If significant differences were present by ANOVA ($p < 0.05$), a least significant differences (LSD) test was performed post hoc. Between group differences were considered

statistically significant when $p < 0.05$. Statistical tests were performed using GraphPad Prism version 7.00 for Windows, (GraphPad Software, La Jolla California USA).

To determine whether the expression of *NAMPT* could predict BPD, we randomly split the human microarray data into 2/3 for training and 1/3 for testing. We fit a single gene logistic regression model with BPD as our dependent variable and *NAMPT* as our independent variable. The area under the receiver operating characteristic curve (AUC) was calculated, wherein 70% was deemed as clinically relevant. The same approach was utilized for the AUC of no BPD against severe BPD. Genomic and human cohort analyses were performed in R v.4.1.0 using the packages GEOquery, limma, DESeq2, pROC, ggpubr, and gt summary.
